# Impact of mobility restriction in COVID-19 superspreading events using agent-based model

**DOI:** 10.1371/journal.pone.0248708

**Published:** 2021-03-18

**Authors:** L. L. Lima, A. P. F. Atman

**Affiliations:** 1 Programa de Pós-Graduação em Modelagem Matemática e Computacional, Centro Federal de Educação Tecnológica de Minas Gerais—CEFET-MG, Belo Horizonte, Minas Gerais, Brazil; 2 Departamento de Física, Centro Federal de Educação Tecnológica de Minas Gerais—CEFET-MG, Belo Horizonte, Minas Gerais, Brazil; 3 National Institute of Science and Technology for Complex Systems—CEFET-MG, Belo Horizonte, Minas Gerais, Brazil; Universidad Nacional de Mar del Plata, ARGENTINA

## Abstract

COVID-19 pandemic is an immediate major public health concern. The search for the understanding of the disease spreading made scientists around the world turn their attention to epidemiological studies. An interesting approach in epidemiological modeling nowadays is to use agent-based models, which allow to consider a heterogeneous population and to evaluate the role of superspreaders in this population. In this work, we implemented an agent-based model using probabilistic cellular automata to simulate SIR (Susceptible-Infected-Recovered) dynamics using COVID-19 infection parameters. Differently to the usual studies, we did not define the superspreaders individuals *a priori*, we only left the agents to execute a random walk along the sites. When two or more agents share the same site, there is a probability to spread the infection if one of them is infected. To evaluate the spreading, we built the transmission network and measured the degree distribution, betweenness, and closeness centrality. The results displayed for different levels of mobility restriction show that the degree reduces as the mobility reduces, but there is an increase of betweenness and closeness for some network nodes. We identified the superspreaders at the end of the simulation, showing the emerging behavior of the model since these individuals were not initially defined. Simulations also showed that the superspreaders are responsible for most of the infection propagation and the impact of personal protective equipment in the spreading of the infection. We believe that this study can bring important insights for the analysis of the disease dynamics and the role of superspreaders, contributing to the understanding of how to manage mobility during a highly infectious pandemic as COVID-19.

## Introduction

SARS-CoV-2 (severe acute respiratory syndrome coronavirus 2) has spread worldwild, being an immediate major public health concern [1]. Governments and research centers around the world have joined efforts to help combat COVID-19, seeking alternative solutions to contain the pandemic. Some countries, like China and New Zealand, have succeeded to control the first wave of the disease with severe restrictions on travel and mobility, along with other actions, as detecting and isolating cases [2–4].

In a scenario of searching for measures to contain disease spreading, epidemiological models can be of great help, becoming a useful tool to assist in decision making. One of the epidemiological parameters frequently found in these models is the basic reproduction number (*R*_0_), which is defined as the mean number of infections generated by an infected individual in a susceptible population. Its value has been estimated between 1.4 and 6.49 for COVID-19 [5].

If we analyze only *R*_0_, values less than 1 indicate that the infection spreading is shrinking and, on the other hand, when *R*_0_ is greater than 1, the infection tends to spread to the entire population [6]. However, *R*_0_ is an average value, and may not be enough to indicate whether the epidemic will continue to spread or not, especially if we consider the occurrence of superspreading events (SSEs): as a mean number, *R*_0_ can distort individual infectiousness [7] and it may not be a good metric when population heterogeneity increases [8]. It may hide the fact that few individuals are normally responsible for transmitting the disease [9].

SSEs are defined as outbreaks where a large number of cases are caused by a small number of infected individuals, i.e., some individuals have high infection capacity [10]. Superspreaders promote a wide spread of the disease [11] and a single case can be responsible for an explosive epidemic when a disease has a high individual variation [7]. Other factors, as transmission mode, contact frequency and duration, and public health interventions, can also impact the occurrence of superspreading events [12]. SSEs can be modeled using a negative binomial distribution and a dispersion parameter *k*[7]. Smaller values of *k* can indicate an over-dispersion, with few individuals being responsible for many infections [9].

Superspreading had special attention in SARS outbreaks in Singapore and China in 2003, since they helped in understanding transmission dynamics [13]. In Singapore, five people caused more than half of the 205 cases (and 163 cases led to zero secondary transmission)[14]. In 2015, in Korea, only 5 cases of MERS originated 154 secondary cases (166 cases led to zero secondary infections)[13, 15]. For COVID-19, one of the most emblematic cases of superspreading at the beginning of the pandemic was the “Patient 31”, who was linked to a cluster with more than 5.000 cases in Daegu, South Korea [16].

In this paper, we aim to use an agent-based model, which allows incorporating spatio-temporal factors and heterogeneity in the population [15], to evaluate the presence of superspreaders in COVID-19 infection scenarios with reduced human mobility. Our analysis was based on measurements made on networks of infected individuals built from simulation models. To model SSEs, some authors usually tag these individuals before the simulations starts, by attributing some characteristics that differentiate superspreaders from other individuals [8, 11, 15, 17, 18]. Here, we are looking for evaluating if a system presents these SSEs features as emergent behavior, identifying if there are superspreaders in the population without inserting SSE characteristics to some individuals *a priori*, and proposing an auxiliary method to identify key-spreaders.

## Materials and methods

Here we present the basic method developed to run the simulations. We designed an agent-based model (ABM) combined with probabilistic cellular automata (CA) to mimic the dynamics of a heterogeneous population within an urban area. One of the characteristics of CA is that they are simple enough for detailed mathematical analysis but also quite sophisticated to be used to study complex phenomena [19]. Our CA consists of a grid of cells over a regular square lattice (*L* × *L* sites, each site corresponding to ∼9*m*^2^) where the simulation takes place. Each agent can assume a finite number of states that are updated synchronously each time step. In this study, each agent can be in one of the following states: Susceptible, Incubated, Asymptomatic Infected, Symptomatic Infected, Infected in the hospital ward, Infected in Intensive Care Unit (ICU), Recovered, and Dead. In case of infection, it is defined *a priori* if the individual will be asymptomatic, have light symptoms, need to go to the hospital, or to die. This choice intends to reproduce the genetic heterogeneity of the population as well the health condition of a given agent.

Time step is equivalent to one hour and agents execute a random walk along the environment, using Moore’s neighborhood and with periodic boundary conditions. When two or more agents are in the same site and one of them is infected, the susceptibles ones have a probability of 80% of becoming infected, except if those individuals are in the infirmary or in the ICU, once in these sites they are considered as isolated. Both symptomatic and asymptomatic can transmit the disease [20]. We do not consider the possibility of reinfection along the period of the simulation run.

To evaluate the impact of personal protective equipment (PPE), we also run simulations where there is a 70% probability that an individual is wearing a mask. In this case, if two individuals are in the same site and not wearing a mask, we keep the 80% probability of infection transmission, but, in the case of one of them is wearing a mask, there is 50% probability of transmission, and just 10% of probability in the case of both are wearing masks.

All simulations begin with only susceptible individuals and we choose randomly one of them to be infected. The period of permanence into a given state follows a Gaussian distribution around the mean intervals shown in Table 1. These mean values were extracted from literature as typical for COVID-19 [21–24].

**Table 1:** Time (days) used as model parameters.

State	Mean	Minimum	Maximum
**Incubation**	5.2	2	12
**Infection (after incubation)**	5.8	3	14
**Infirmary (after infection, if hospitalized)**	10.5	7	14
**ICU (after infirmary, if goes to ICU)**	17.5	14	21

To analyze mobility restriction, the model considers a reduction in the individual displacements by a percentage (0% or no restriction, 10%, 20%, 30%, 40%, 50%, 60%, 70%, 80%, and 90%). High mobility restriction values mean that the agents move less. For example, if an individual takes approximately 24 steps by day in a normal situation (0% restriction), with 50% restriction it will take approximately 12 steps. With an 80% restriction, the displacements will be reduced to 4 or 5 steps by day. We apply the Monte Carlo method (64-bit Xorshift algorithm [25]) to implement the randomness in the model. It worth noting that the algorithm does not prevent the agents from meeting each other, but since everyone is displacing less, the chance of encounters is reduced.

**Fig 1:**
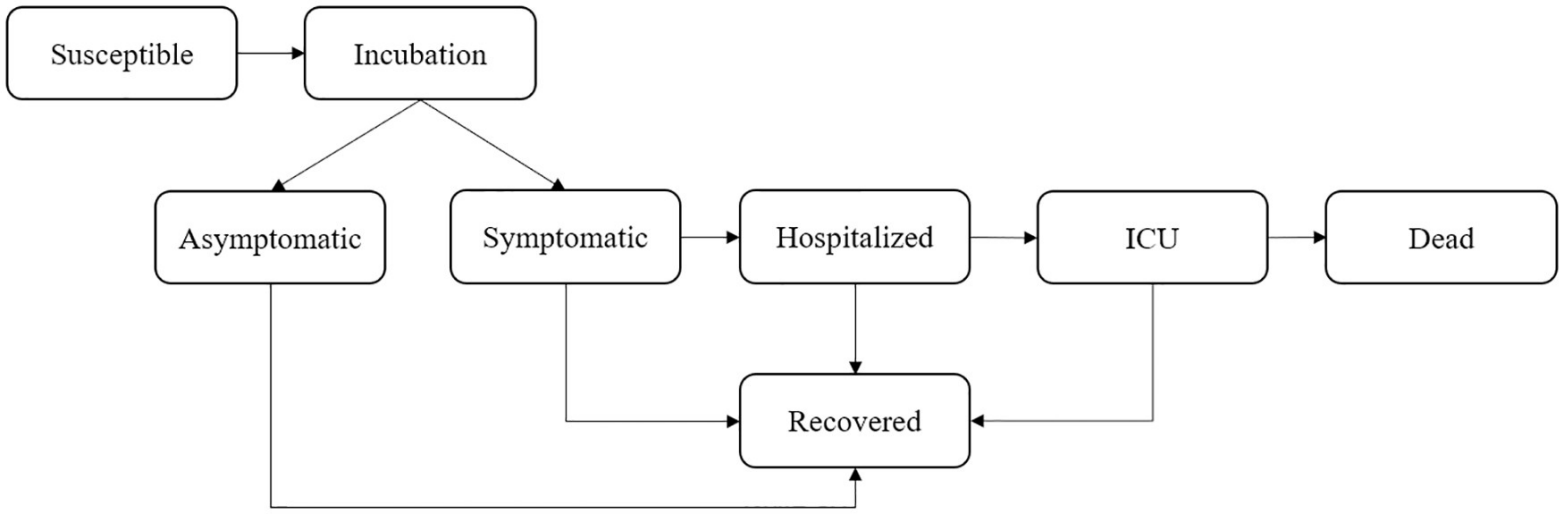
Dynamics of the model: An infected individual can be symptomatic or asymptomatic. Symptomatic individuals can go to the infirmary and, eventually, to the ICU.

For this work, we used *L* = 250 and 10000 individuals. Risk population is distributed according the following probabilities (considering the case of the individual be infected): 60% will become symptomatic [20]; 21% will need to go to a hospital [26]; 5% will need ICU [26] and 2% of death rate [26], as Fig 1.

We calculate the basic number of reproduction in two ways: a) by dividing the number of new infections per day by the actual number of individuals responsible for those infections (*R*_0_); b) by dividing the number of new infections per day by the total number of infected (and potential transmitters of the disease) on that day ($R_{0}^{*}$). The aim for these different definitions is to compare the simulation results with the real statistics of the infection furnished by the public health institutions which can not access the actual data of transmission among individuals.

### Network analysis

By treating the results, it was possible to build a directed graph from the originally infected individual towards the subsequent ones, allowing to visualize the transmission network of the disease. It was done recording the infection chain from individual to individual. Agents not infected were not considered in the graph.

Each infected agent is considered as a vertex of the network and we calculated the degree of a vertex *v* as the number of links connected to *v*. We called this value as *deg*(*v*). We consider only the out-degree, that is, how many people does an infected individual transmit the disease. So, the degree distribution measured in this work only considers out-degree connections.

The betweenness centrality of a node *v*, *B*(*v*), is given by Eq 1:

$$\begin{array}{c} B\left(v\right)=\sum_{s\neq v\neq t}\frac{\sigma_{st}\left(v\right)}{\sigma_{st}}\;, \end{array}$$ (1)

where *σ*_*st*_ is the total number of shortest paths from node *s* to the node *t* and *σ*_*st*_(*v*) is the number of these paths that pass through *v*. We performed a normalization by dividing *B*(*v*) by (*N* − 1)(*N* − 2), since the graph is directed. *N* is the number of nodes (vertices) of the graph.

We also calculate the closeness centrality, which is given by *C*(*v*) in Eq 2.

$$\begin{array}{c} C\left(v\right)=\frac{N-1}{\sum_{u}d\left(v,u\right)}\;, \end{array}$$ (2)

Note that the Eq 2 is normalized due the factor (*N* − 1) in numerator. In this equation, *d*(*v*, *u*) is the distance (number of nodes) in the shortest path between vertices *v* and *u*.

## Results

In this section, we present the results obtained for the infection spreading considering several percentages of mobility restriction and the impact of the use of personal protective equipment. All the curves correspond to averages over 30 simulation runs.

### Mobility restriction scenarios

**Fig 2:**
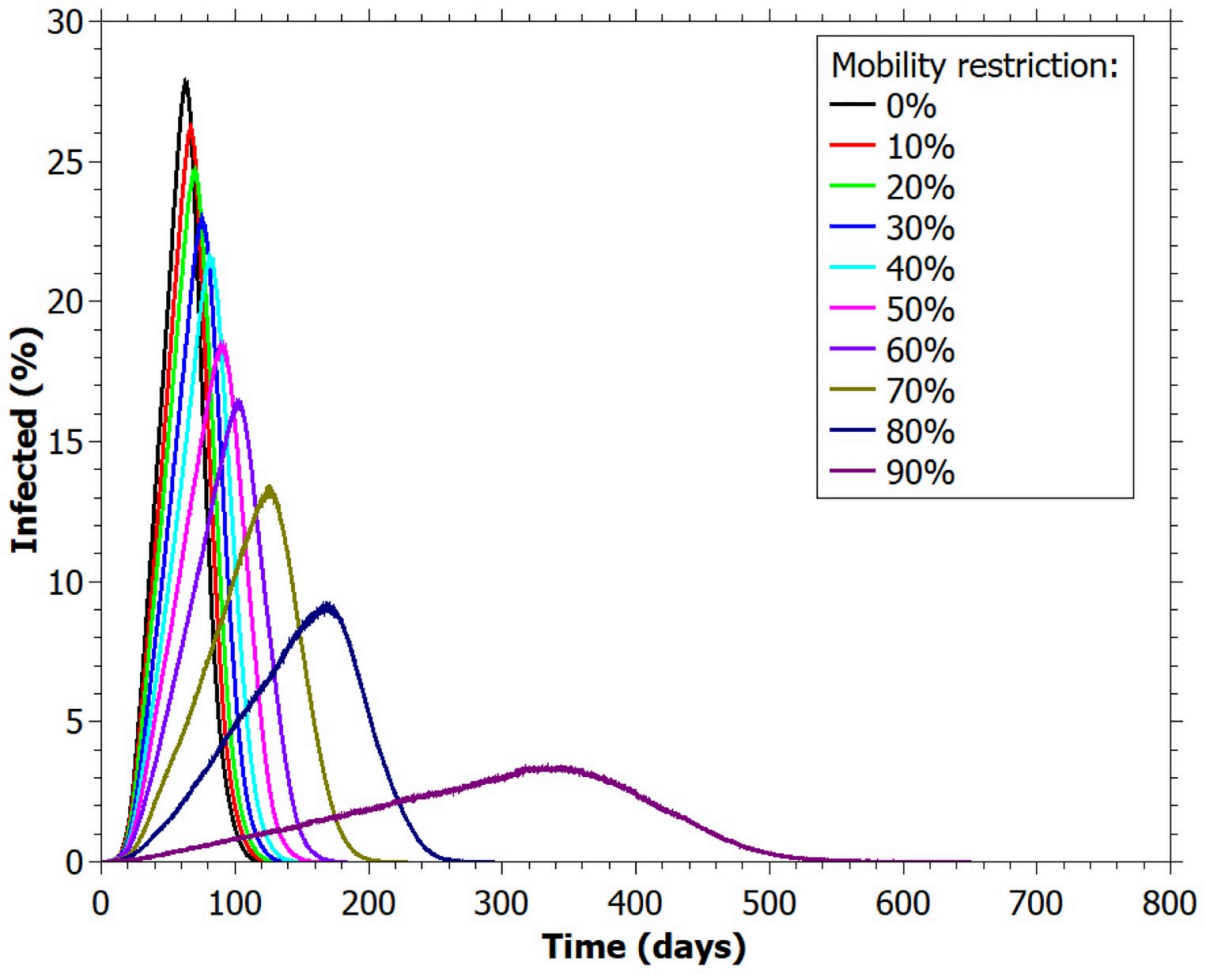
Percentage of infected agents for different values of mobility restriction.

In Fig 2 we observed a remarkable difference among the curves of the number of infected agents in function of mobility restriction. The results suggest that decreasing lightly the mobility does not have a strong impact in spreading infection, as observed in 10%, 20%, 30%, and 40% curves. The most effective result for flattening the infection curve was observed in the 90% of restriction. Reducing the mobility by 70% and 80% also showed a considerable flattening when compared with scenario of no restriction (0%).

**Fig 3:**
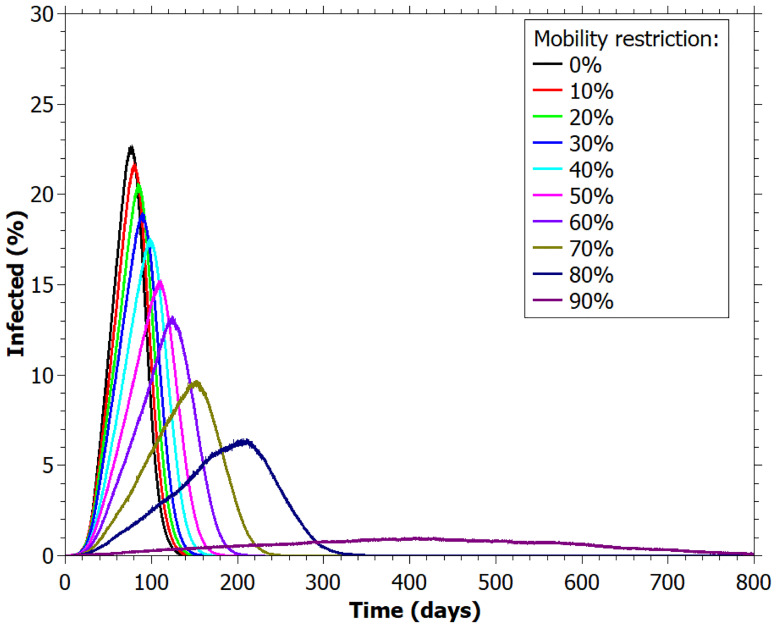
Percentage of infected agents for different values of mobility restriction for 70% of individuals wearing masks.

In Fig 3 we visualize the impact of PPE (personal protective equipment) in the flattening of the infection curve. Clearly, the PPE is effective to retard the infection spreading. With 70% of humans wearing a mask, we see a reduction in peak curves for all the restrictions values, compared to cases in which people do not wear it (Fig 2).

**Fig 4:**
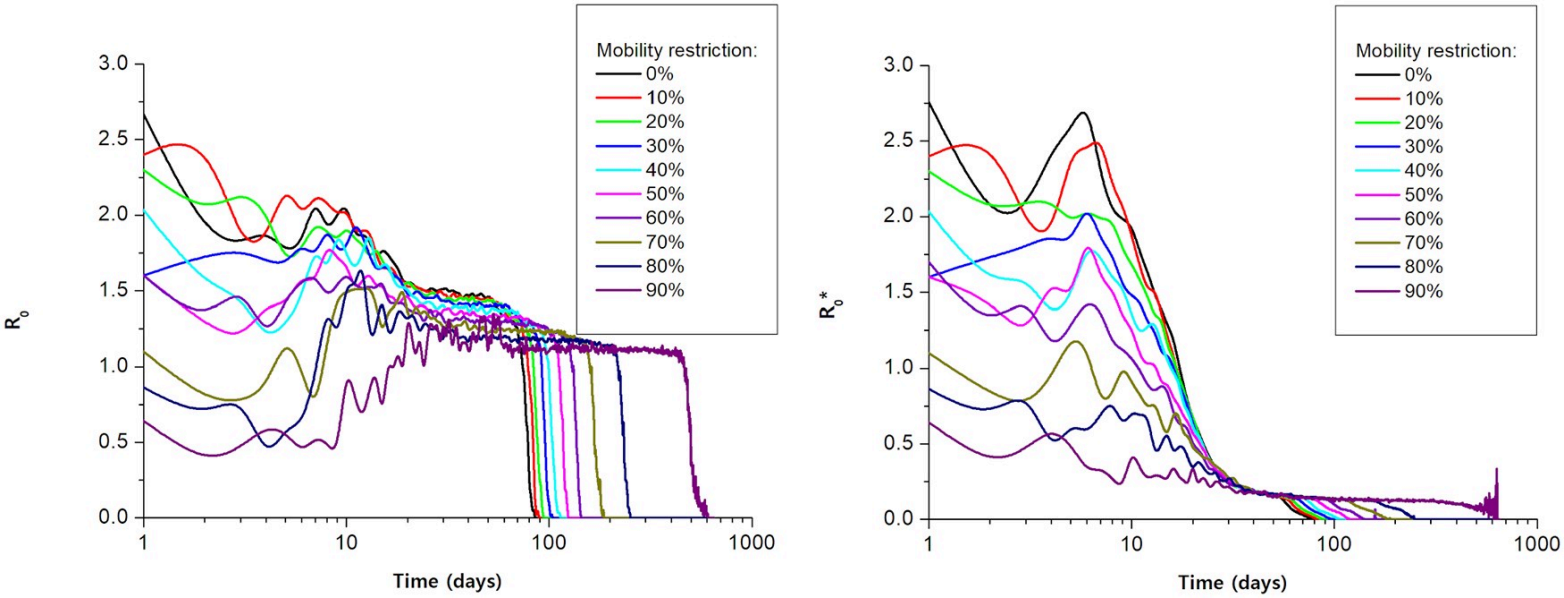
(a) Number of new infections per day divided by the actual number of individuals responsible for those infections for different values of mobility restriction (*R*_0_). (b) Number of new infections per day by the total number of infected on that day for different values of mobility restriction ($R_{0}^{*}$).

Fig 4 shows the results for *R*_0_ and $R_{0}^{*}$. It was observed that the greater the mobility restriction, the lower the value of these indexes, mainly at the beginning of the disease spreading. As time goes by and, consequently, fewer individuals remain susceptible, the *R*_0_ value tends to be close to 1 in Fig 4a, that is, one person infects one person, and $R_{0}^{*}$ lower than 1 (Fig 4b).

In Table 2 it is possible to notice a delay of approximately 267 days to reach the peak of the pandemic and approximately 24.5% less of infected individuals comparing the more restrictive scenario (90%) with the no restriction case (0%). This flattening is also reflected in the percentage of the population hospitalized in ICUs (0.35% compared with 2.87%) at the peak. This represents a difference of about 252 ICU beds for every 10000 inhabitants and demonstrates how the mobility restrictions are fundamental to avoid the collapse of the health system.

**Table 2:** Maximum infected and number of ICU beds required every 10000 individuals for each percentage of mobility restriction. Simulations without PPE.

Mobility restriction	Max. infected(%)	Time (days) max. inf	Time (days) max. ICU	Max. ICU/10000 individuals	Total of infected (%)
**0%**	27.80±0.88	63.09±1.95	83.17±2.61	287±16	99.99±0.01
**10%**	26.11±0.87	67.125±2.30	86.13±2.83	266±22	99.99±0.01
**20%**	26.64±0.89	75.33±2.41	89.88±3.56	251±22	99.98±0.01
**30%**	22.883±0.81	75.33±2.47	94.79±3.55	230±21	99.98±0.01
**40%**	21.50±1.08	81.951±2.60	102.21±4.09	217±13	99.97±0.01
**50%**	18.38±0.57	90.17±3.81	109.25±4.88	189±13	99.94±0.02
**60%**	16.36±0.82	102.83±4.41	122.17±5.44	162±11	99.86±0.04
**70%**	13.20±0.73	124.88±7.23	148.17±9.27	133±9	99.58±0.06
**80%**	9.08±0.57	168.42±8.43	189.04±12.19	93±10	98.22±0.15
**90%**	3.34±0.43	330.46±38.09	355.96±47.06	35±6	84.41±0.95

An interesting highlight is that practically all individuals who became infected in the simulations run up to 60% mobility restriction. For 70% of reduction, the non-infected rate is about 0.4% and it increases up to 16% for the case of 90% restriction (Table 2).

### Network analysis

**Fig 5:**
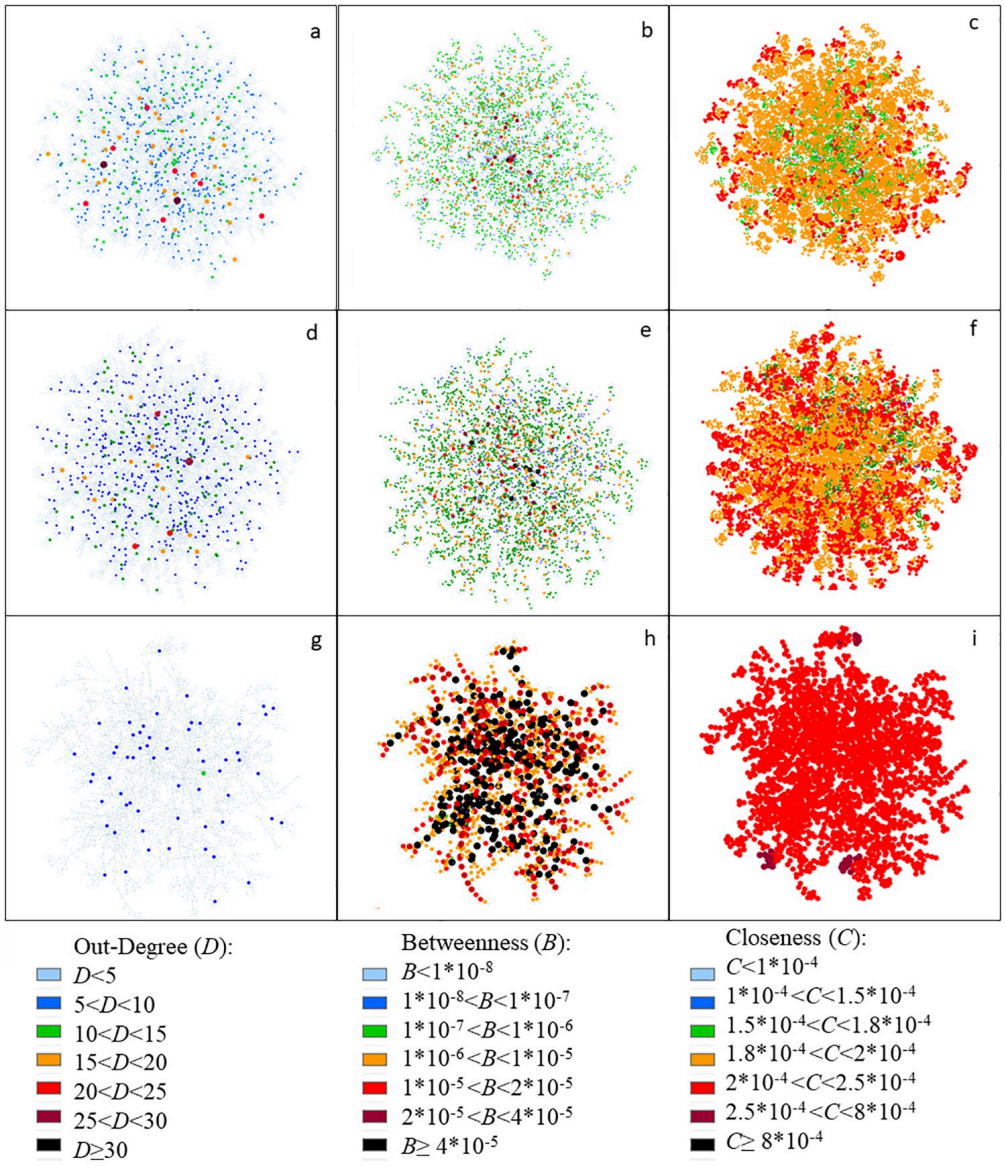
Example of transmission networks generated at the time step corresponding to the maximum of infected agents in each mobility restriction scenario, without PPE, colored according to out-degree value, betweenness and closeness centrality: (a) Out-degree and 0% of mobility restriction; (b) Betweenness centrality and 0% of mobility restriction; (c) Closeness centrality and 0% of mobility restriction; (d) Out-degree and 40% of mobility restriction; (e) Betweenness centrality and 40% of mobility restriction 40%; (f) Closeness centrality and 40% of mobility restriction; (g) Out-degree and 90% of mobility restriction; (h) Betweenness centrality and 90% of mobility restriction; (i) Closeness centrality and 90% of mobility restriction.

Fig 5 shows the infection network for 0%, 40% and 90% mobility restriction level. Graphs were generated at the time step corresponding to the peak of the infection curve (Fig 2) and colored according to different values of degree, betweenneess and closeness centrality.

**Fig 6:**
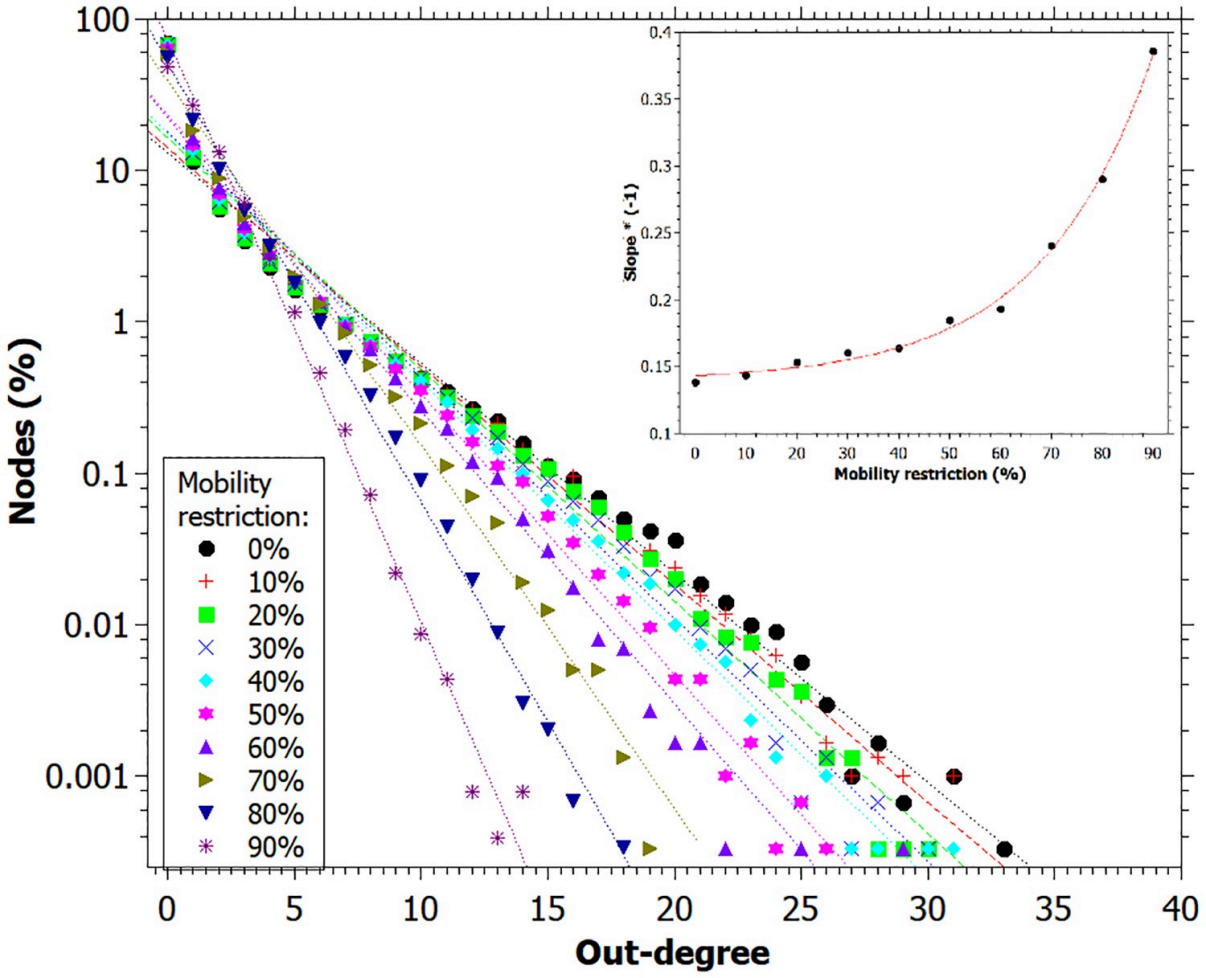
Out-degree distribution for each mobility restriction percentage considered. Lines corresponds to exponential fitting. Inset of the fitting parameters versus the percentage of restriction mobility. Data registred at the end of the simulation.

In Fig 6 are shown the degree distribution for each value of mobility restriction at the end of simulation. There is a striking shrinking of the degree distribution for increasing mobility restriction, which means that the average value for *R*_0_ should decrease, as verified. This observation corroborates the fact that a large number of people do not transmit the disease to anyone, while a small number of individuals are responsible for a large number of infections, characterizing superspreading dynamics.

The dependence of the fitting parameters versus the percentage of restriction mobility is shown in the inset of Fig 6. It is clear that for small values of mobility restriction, this dependence is quite subtle, which corroborates the observation made previously that the spreading dynamics is almost independent of the mobility restriction for lower levels. A significant flattening of the curves is observed only for restrictions above 70%.

**Fig 7:**
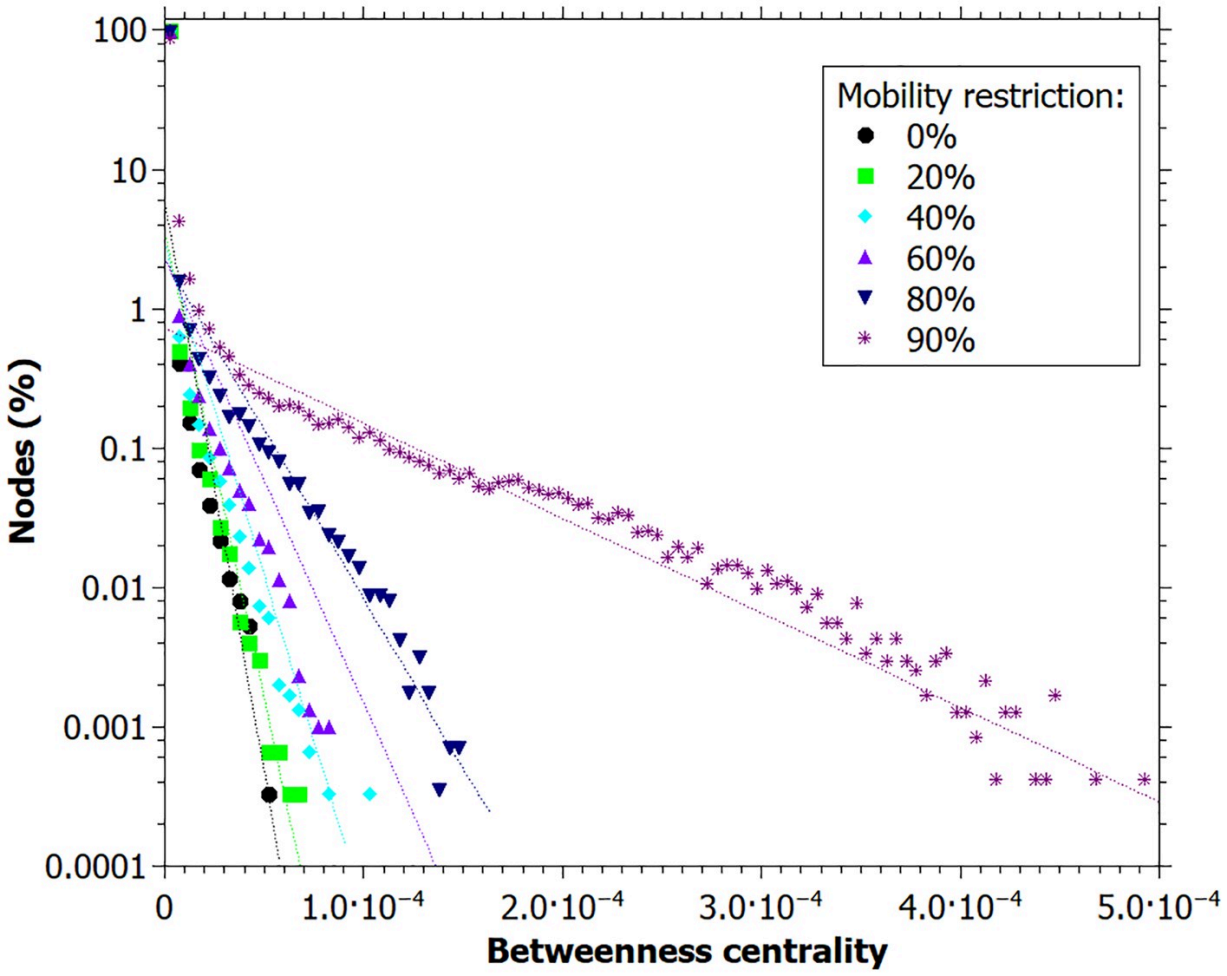
Betweenness centrality distribution of each mobility restriction value. Lines are just guides for the eyes. Data registred at the end of the simulation.

**Fig 8:**
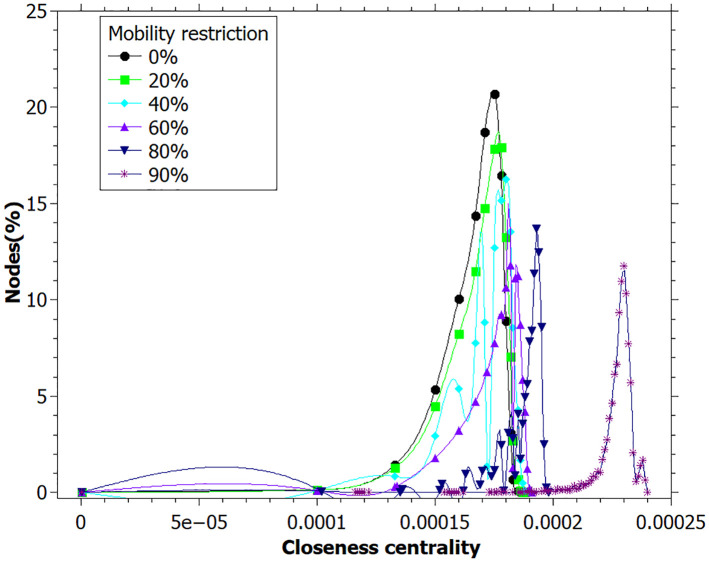
Closeness centrality distribution for each mobility restriction value. Lines are just guides for the eyes. Data registred at the end of the simulation.

We can see the distributions of betweenness and closeness centralities at the end of simulation in Figs 7 and 8. Both measures present a positive correlation with higher mobility restriction values. There are a large number of individuals with a betweenness centrality value close to zero, especially for low mobility restriction values. High values of betweenness can be associated with key-spreaders since it means that the agent connects several infected individuals.

**Fig 9:**
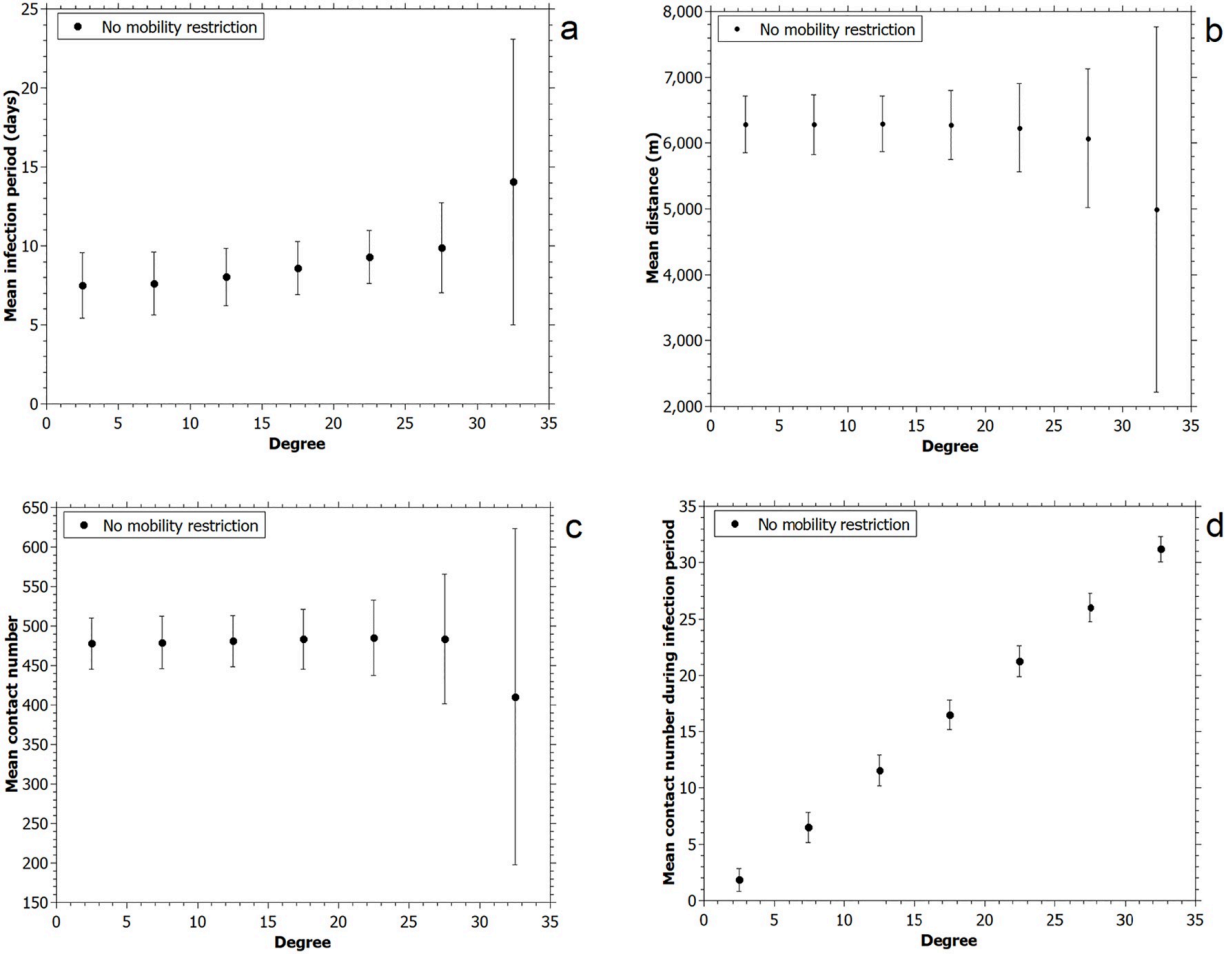
(a) infection period; (b) travelled distance during simulation; (c) number of contacts during whole simulation and (d) number of contacts during infection period. All distributions in function of degree.

In order to evaluate which intrinsic features characterize superspreaders, we compare the degree of the individuals with four infection parameters: distance traveled, infection period, number of contacts along the simulation, and number of contacts during the infection period. Pearson correlation test furnished, respectively: 0.0007; 0.05; 0.02 and 1 for no mobility restriction scenario, as shown in distributions in Fig 9. As expected, test showed that there is a strong positive correlation between degree and number of contacts during the infection period, but no marked dependence with the other parameters. The results for high mobility restrictions are similar: 0.12; 0.021 and 1, for infection period, number of contacts along the simulation and number of contacts during the infection period, respectively, for mobility restriction of 80%.

**Fig 10:**
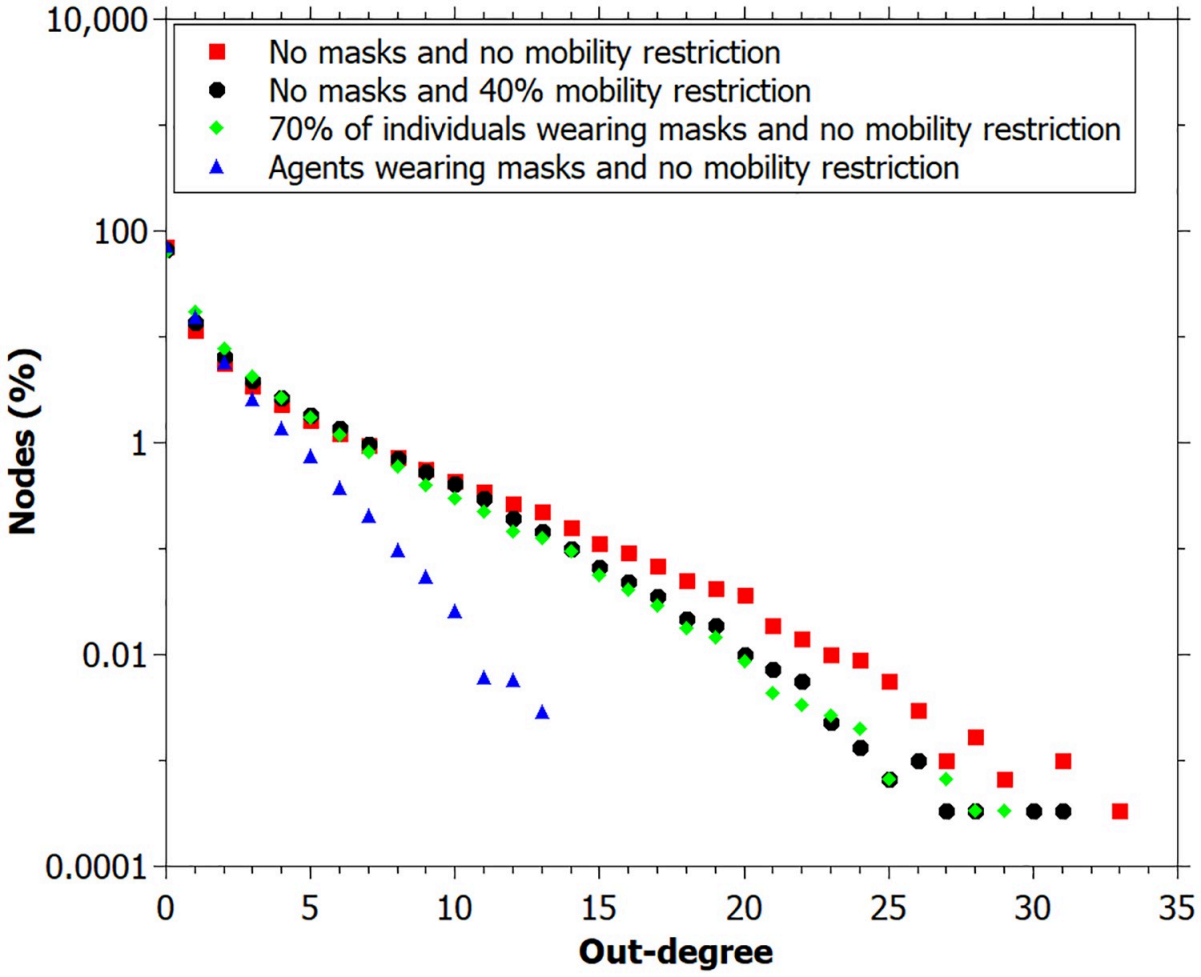
Out-degree measured for simulations: No masks and no mobility restriction; no masks and 40% mobility restriction; individuals wearing masks and for all individuals. Data registred at the end of the simulation.

**Fig 11:**
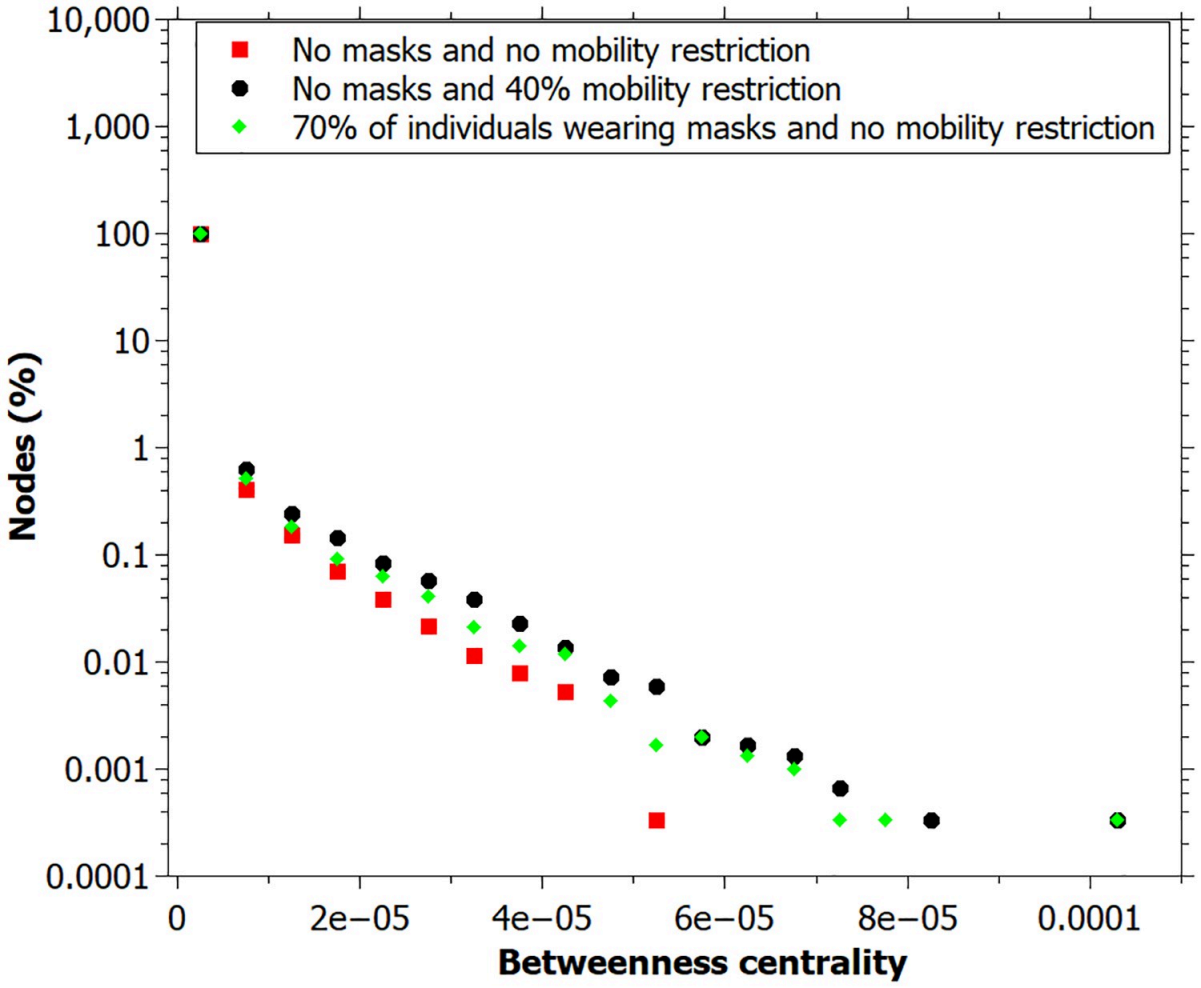
Betweenness centrality measured for simulation: No masks and no mobility restriction; no masks and 40% mobility restriction; 70% wearing masks and no mobility restriction. Data registred at the end of the simulation.

**Fig 12:**
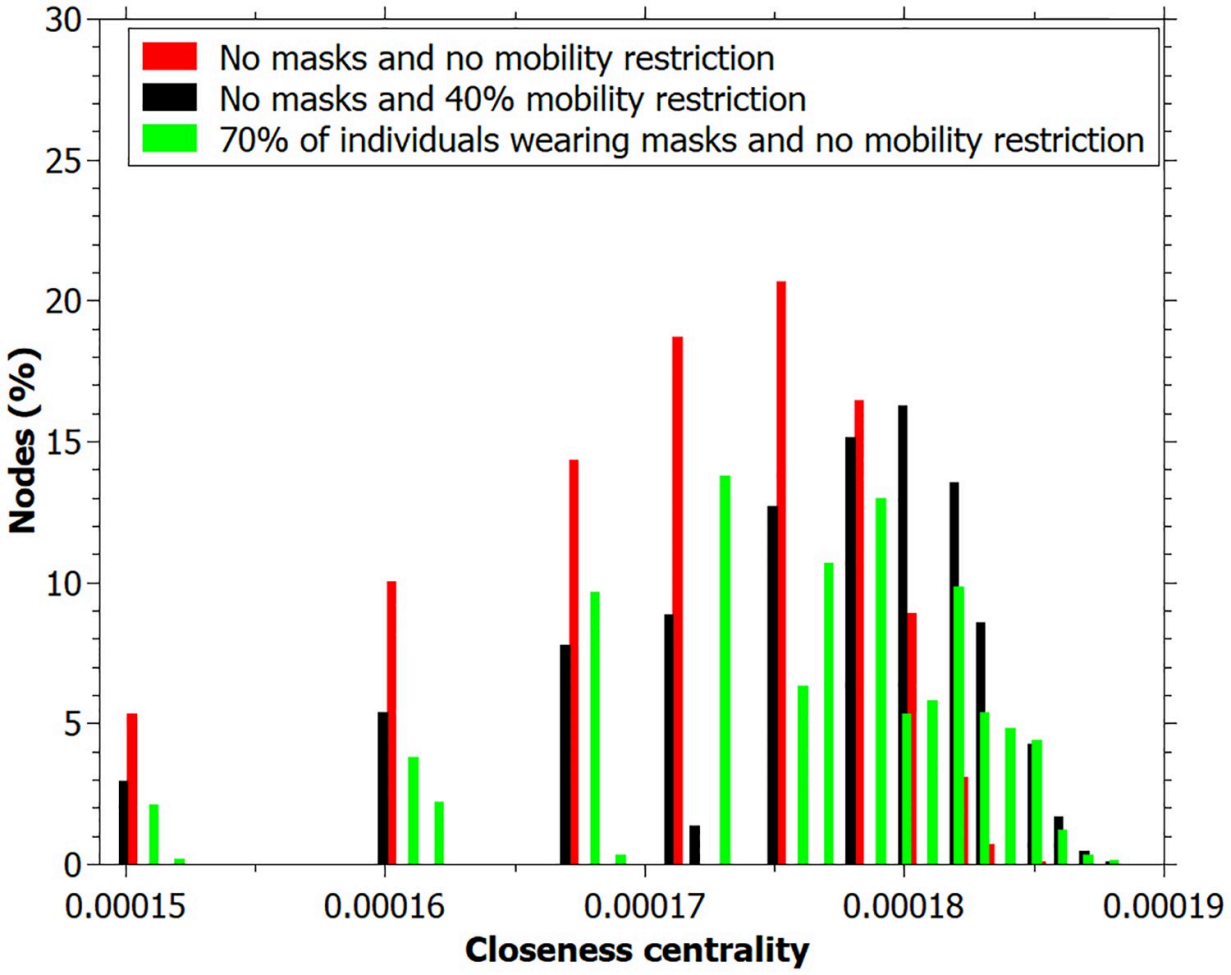
Closeness centrality measured for simulation: No masks and no mobility restriction; no masks and 40% mobility restriction; 70% wearing masks and no mobility restriction. Data registred at the end of the simulation.

We also can see that the out-degree, betweenness and closeness centrality values at the end of simulation, for the case with 70% of individuals wearing masks are very similar to the simulation with no masks and 40% of mobility restriction (Figs 10–12).

Concerning the relation of using mask and degree distribution, we found a Pearson’s correlation coefficient of -0.38, indicating that individuals wearing masks tend to have a lower degree than those who do not, as seen in Fig 10, which shows that the higher degree values are from the agents who do not wear a mask. This result corroborates the practice of wearing masks to avoid the infection spreading [27].

## Discussion

Fig 2 and Table 2 show that increasing the mobility restriction leads to a flattening of the infected curve, which consequently reduces the ICU bed occupation, especially for situations near to lockdown, as 70-90% mobility restriction. However, it is almost impossible to maintain a long time in lockdown, not only due to socio-economic impact [28], but also due to consequences for the physical and mental health of the population [29, 30].

We also notice that there is not big difference between the Fig 3 curves for low and medium variation in mobility reduction, suggesting that although the use of masks may contribute to the reduction of the spread of the disease, other combined non-pharmaceutical interventions may be necessary to fight the virus spreading, as in isolation, quarantine, contact tracing and physical distancing [31]. Thus, it is necessary that public health organizations have a policy that involves several actions. For a disease whose effects are not fully yet known, as for COVID-19, with a fast initial spreading to different countries, characterizing a pandemic, establish severe mobility restrictions is a good initial strategy to give time for governments avoid the collapse of health institutions. Indeed, it was verified by the results in Table 2 that higher mobility restriction values led to fewer individuals to be hospitalized simultaneously.

Fig 4 shows evidence of how the different definitions of basic reproduction number impact the evaluation of pandemics propagation. Firstly, it worth noting that both definitions display similar overall behaviors: after some initial oscillations, there is a plateau for intermediate times and a decrease marking the end of the infection. However, $R_{0}^{*}$ values are significantly smaller than those for *R*_0_, as one can observe in the plateau values. The marked difference in the values of *R*_0_ and $R_{0}^{*}$ can be understood in terms of the superspreaders. While *R*_0_ plateau is above unity, $R_{0}^{*}$ saturates at values quite below unity, indicating that most part of the infected agents do not transmit the infection.

In our simulations, 70% of the individuals did not infect anyone and 13% were responsible for 77% of the infections. In comparison, a COVID-19 study in Hong Kong showed that 69% of infected did not spread the disease, while 17-19% of infectious individuals were responsible for 80% of all transmission events [32].

This conclusion corroborates other studies that have already shown that the existence of superspreaders can significantly impact the pattern of outbreaks and, consequently, it is necessary to be careful when interpreting epidemiological parameters as *R*_0_[8]. This indicates that the methodology to calculate the basic reproduction number should be carefully analyzed to avoid underestimating the power of the infection transmission.

An important feature presented in other studies of superspreading is that the heterogeneity is assigned *a priori* to the individuals, as a longer infectious period [8], a higher level of infectivity [8, 11, 15], a high number of contacts [11, 15, 17, 18] or a longer period out of isolation [33]. Here, we only implemented Gaussian distribution in the infection duration, but we did not establish larger values for the superspreaders. Instead, we analyzed the results to verify whether it would possibly to identify the superspreaders based on these parameters. In other words, the superspreaders were not defined *a priori*, but they were identified after the simulation ran based on the statistics of the infection. Their existence became clear by looking at the degree distribution (Fig 6), in which is possible to observe large degree individuals up to value 34, that is, one individual transmitted the disease directly to 34 other individuals.

Besides, with increasing mobility restriction, the maximum degree of the superspreaders tends to decrease, but the spreading dynamics remains the same. In Fig 6, the distribution exhibits heavy tails, as observed for the SARS epidemic in Singapore in 2003 [14]. In this way, some researchers affirm that superspreading is a typical feature of disease spreading [7] and that data from infectious diseases tend to have a variance greater than the mean, that is, they tend to be over dispersed [14].

In Fig 9 it is not clear if there is a correlation for infection period, traveled distance, and number of contacts during simulations, since high degree values have a higher error bar. However, it is evident that there is a positive correlation between the number of contacts during the infection period and degree. These results highlight the importance of isolating infected individuals, reducing their contacts during the period they can spread the disease. In practice, contact testing and tracing are essential to identify those infected, especially for asymptomatic.

Fig 5 shows that the greater the mobility restriction, the higher the closeness and betweenness centrality values and the lower the degree values for each network node at the peak of infection. Figs 7 and 8 together with the out-degree distribution (Fig 6) also evidence a change in the network structure due to the mobility restriction (at the end of simulation). In Fig 7, the higher the restriction, the higher the maximum values of betweenness centrality. Since betweenness is related to how many times a vertex appears in shorter paths, the increase of this measure indicates that the infection spreads at a slower rate, once the route of infection become more concentrated in the superspreaders. On the other hand, when there is no restriction, the transmission is made by more people, making the infection spreading be more distributed among the agents, leading to smaller values of betweenness. This enhancement of spreaders’s role as mobility decreases is not captured by the degree distribution, and it is an original contribution of this study. It is possible to identify key-spreaders that do not have a very high degree but are essential to keep the transmission active even with reduced mobility.

A similar interpretation can be inferred for the closeness centrality (Fig 8). For higher values of mobility restriction, there is a small number of individuals with large closeness values, indicating that they transmit the disease more efficiently through the network. Otherwise, for those networks built with a lower mobility restriction, a large number of individuals presents intermediate closeness values. This agrees with the reasoning that there are more agents infected transmitting the disease at the same time.

It is important to notice that the results of the simulation with individuals wearing masks furnished a lower degree distribution since the probability of transmission with masks is lower, and these individuals have a lower degree than the others (Fig 10). Betweenness and closeness centrality values are similar to that found for simulation with no masks and 40% of mobility restriction, indicating that the act of wearing a mask has an equivalent effect to restrict mobility by 40% at the simulation level (Figs 11 and 12).

In the scenarios with higher values of mobility restriction, we verify the existence of individuals with high values of betweenness and closeness (Figs 7 and 8), highlighting the importance of testing and trace infected people during a pandemic. By isolating these potential disseminators, it is possible to retard the massive spread of the disease and allow more human circulation in the cities. This consequently contributes to reducing the social and economic impacts of the pandemics.

## Conclusion

In general, the simulation showed that reducing mobility is effective in flattening the infection curve. Also, agents wearing a mask spread the infection to a smaller number of people, contributing to the flattening of the curve even in a scenario without mobility restriction.

Regarding superspreading, simulation analysis evidence that this is an emergent feature of epidemics, and it could be quantified by network measures as out-degree distribution, betweenness and closeness centrality, without the need to artificially introduce this kind of agent. Besides, there is a reduction of the degree distribution as the mobility restriction increases together with an increment of betweenness and closeness centralities values. It reveals the most prominent role of some individuals to spread the disease, as key-spreaders. Testing and tracing contacts is essential to make mobility restrictions more flexible, in addition to other preventive measures as wear personal protective equipment, allowing infected individuals to remain in isolation and do not become superspreaders.

Controlling a pandemic requires high-stakes decisions that involve different factors at different levels of government and public health organizations. It is necessary to use appropriate tools to support these decisions, which highlights the need for investment in science. Epidemics that came before COVID-19 showed the need for governments to be prepared to face the adversities of a disease that can affect the entire world. In this way, understanding the role of the players in infection propagation is extremely important for making prediction models and for establishing disease control strategies.
